# The role of self-advocacy and self-determination in positive adjustment for autistic adolescents and young adults: a mini-review

**DOI:** 10.3389/frcha.2025.1542543

**Published:** 2025-05-02

**Authors:** Daniele C. Martino, Alexa Brantley, Angela Scarpa

**Affiliations:** ^1^ Department of Psychology, Virginia Tech, Blacksburg, VA, United States; ^2^ Virginia Tech Autism Clinic & Center for Autism Research, Virginia Tech, Blacksburg, VA, United States; ^3^ ORAU-Directed Research and Development, Oak Ridge Associated Universities, Oak Ridge, TN, United States

**Keywords:** autism, self-advocacy, self-determination, adolescence, young adulthood, neurodiversity

## Abstract

Autistic individuals have an increased likelihood for negative adjustment relative to their peers, often as a result of adverse experiences. Consistent with the biosocial model of resilience and the growing neurodiversity movement, identifying factors that may contribute to positive outcomes among autistic individuals is an urgent research priority. The present review explores the existing literature on the role of self-advocacy and self-determination in promoting positive adjustment for autistic adolescents and young adults. Findings point to encouraging associations of self-advocacy and self-determination with various adjustment outcomes, including educational and employment outcomes, socialization, relationship development, identity development, self-concept, and quality of life. Implications are discussed, including limitations, directions for future research, and considerations for designing interventions that support autistic individuals to act with the agency and autonomy they desire.

## 1 Introduction

Autism spectrum disorder (ASD) is a neurodevelopmental condition characterized by differences in social interaction and communication and the presence of restricted interests and repetitive patterns of behavior (1). Autistic youth and young adults are more likely to experience co-occurring mental health conditions (2) and higher rates of stress, trauma, and social adversity relative to their non-autistic peers (3, 4). As such, identifying factors that may contribute to positive adjustment for this population is an important research priority.

According to the Biosocial Model for Resilience proposed by Scarpa et al. (5), variations in neurological functioning interact with social environments to impact resilience in autistic youth, which is defined as avoidance of psychopathology and achievement of life satisfaction, adaptive functioning, and positive well-being and quality of life after experiencing obstacles or stressors. This definition of resilience emphasizes the identification of specific skills and strengths of autistic individuals that may mitigate the negative impact of risk factors, enhance protective mechanisms, and contribute to adaptive functioning. This contrasts prior conceptualizations of risk and resilience applied to autism that have taken a deficits-based approach; that is, one that focuses exclusively on risk and poor outcomes, rather than strengths and skills that contribute to positive outcomes (6). Further, the biosocial framework is consistent with the concept of neurodiversity, which posits that the societal reactions and environments that neurodivergent people experience can contribute to their unique strengths and challenges, and thus neural differences should not be equated to deficits (7).

Two factors that appear to contribute to positive outcomes among autistic youth are self-advocacy (SA) and self-determination (SD). While related, SA and SD are two distinct constructs that have been linked with promoting adjustment via a focus on processes within the individual (8, 9).

SA involves a person understanding themselves, their rights, and their needs, and communicating that understanding (10). Test et al. (11) developed a similar conceptual framework that defines SA as knowledge of the self, knowledge of rights, communication, and leadership. The construct of SD was originally introduced by Deci and Ryan (12) in their proposal of self-determination theory (SDT), which recognizes three psychological needs that are integral to adjustment, motivation, and overall psychological well-being: autonomy, competence, and relatedness. According to the functional theory of SD (13), SD includes four volitional functions that allow a person to act as the primary causal agent in their own life and to maintain or improve their quality of life. These four characteristics include the individual acting in a way that is autonomous, self-regulated, psychologically empowered, and self-realizing.

The functional theory of SD has been applied to individuals with disabilities to emphasize their inherent capability to act in a self-determined manner. Consistent with this view, SD is considered a fundamental right for all people irrespective of disability status (14) and individuals with varied cognitive abilities, including intellectual disability, are capable of being self-determined (15). A qualitative study by Thompson-Hodgetts et al. (16) found that autistic participants emphasized the importance of having influence over what happens in their lives and having support and access to opportunities to make decisions without unnecessary control from others. Results of this study support the notion that SD involves *capacity* to be self-determined as well as having *opportunities* to be self-determined, the latter of which the authors emphasize autistic individuals often lack due to stigma and prejudice. The findings of this study support SD learning theory, which focuses on the process of becoming self-determined and states that SD capacity will not occur without opportunities to become self-determined (17). These conceptualizations of SD are also supported by autistic self-advocates who are part of the *I'm Determined* Project (18). For example, one young adult self-advocate defined autonomy, a central component of SD, as “the ability to act independently and make your own decisions … you are in control of your own life choices”.

SA and SD have become particularly salient within the autistic community with the rise of the neurodiversity movement, which recognizes autism as an essential aspect of a person and a valid way of being (19) and opposes the idea that autistic individuals need to change their behavior to become “less autistic”, or to act in alignment with neurotypical standards of behavior (20). Both SA and SD also are core features of the social model of disability, which, consistent with the neurodiversity movement, emphasizes that disability is not a flaw or defect of the individual but rather arises from the interaction between a “non-standard” individual and a larger society that is unaccommodating (21). The social model of disability supports the idea that individuals with disabilities can advocate for themselves, larger policies, and make changes in their lives through SA and SD.

From a developmental perspective, SA and SD have been identified as important self-processes that develop across the lifespan (22). However, the development of SA and SD may be most important during adolescence and into young adulthood as these are developmental periods noted for autonomy, agency, and identity development (23). Further, these constructs may be particularly relevant in adolescence and into young adulthood for autistic individuals, as the transition to adulthood is seen as a salient transitional period that is marked by poor postsecondary employment and education outcomes following high school for this population (24).

## 2 The present review: rationale and purpose

SA and SD have both been linked with positive adjustment, such as improved quality of life, life satisfaction, communication, and educational and employment outcomes for individuals with intellectual disability, learning disorders, and other high-incidence disabilities (25–28). However, SA and SD are considerably less researched among autistic individuals (29, 30), and this dearth of research is even more pronounced for autistic adolescents and young adults. Together, the potential impact that enhanced SA and SD may have as part of biosocial resilience for autistic individuals in this developmental period is a valuable area to explore alongside the growing neurodiversity movement, yet it is significantly under-researched. The purpose of this mini-review is to explore the existing empirical literature regarding SA and SD and their impact on adjustment outcomes of autistic adolescents and young adults. The term adjustment refers to one's capability to adapt to and meet the demands of the environment, which vary based on developmental stage (e.g., family, school settings; 31). In this mini-review, adjustment is explored broadly with the intent of capturing a range of outcomes that highlight the existing landscape of research in this area.

## 3 Search strategy

A search was completed in Google Scholar in April 2024, using six search terms in different combinations: self-advocacy, self-determination, autism, adolescents, young adults, and positive outcomes. The search included articles published through Spring 2024. Articles were included if they presented an empirical study that assessed SA and/or SD (e.g., either via a measure operationalizing SA and/or SD or an intervention that aimed to foster SA and/or SD skills) related to one or more positive adjustment outcomes and were available in English. Articles are grouped by those discussing SA followed by SD, with one article including both constructs. In this case, the article was discussed in both sections. A complete list of articles included in this review is presented in Table 1. This manuscript was reviewed by the Virginia Tech Center for Autism Research Self-Advocate Autistic Advisory Committee (SAAC) during a regular meeting, with member feedback incorporated into the final version. SAAC members are paid for their work and meet quarterly to offer input on ongoing Center research and programming.

**Table 1:** Sample demographics and key features of included articles.

Study	n	Age group/range	Sample description	Construct	SA/SD measures	Intervention	Outcome variables
Barnard-Brak & Fearon (8)	1,019	Adolescents	Diagnosed with ASD	SA	Combination of teacher and school program surveys asking how well the student asks for what they need	N/A	IEP participation
Bethune (29)	3	High school Students (*Range*_age_ = 16–19)	Diagnosis of mild autism in accordance with the state/federal definition and met DSM-5 definition of Level I or Level II severity criteria as interpreted by Autism Speaks for verbal and non-verbal communication	SA	N/A	The Self-Advocacy and Conflict Resolution training: Strategies for the classroom accommodation request	Knowledge of the skill to request and negotiate academic accommodations
Lei & Russell (9)	18	University students (*M*_age_ = 20.94)	Self-reported formal autism diagnosis from a clinical professional and eligible to access autism specific support on campus	SD	Qualitative interview guide measuring autonomy, competence and relatedness	N/A	University experiences specific to academics, daily living, and socialization
Myers, (33)	6	Adolescents (*Range*_age_ = 11–16)	Diagnosed with high functioning autism or Asperger's syndrome, had awareness of their diagnosis and an understanding of what it means, and able to communicate verbally	SA, SD	Arc's SD Scale for Adolescents	Students Teaching About Autism Reality curriculum	Self-concept/self-esteem; Friendship development and closeness
Pearlman-Avnion et al. (32)	3	Young adults (*Range*_age_ = 21–24)	Diagnosed with high functioning autism or an ASD	SA	N/A	“Avnei Derech” (Milestones) preparatory program	Educational and employment attainment; Identity exploration; Relationship maintenance
White et al. (39)	30	Young adults (*Range*_age_ = 18–29)	Diagnosed with an ASD and no intellectual disability diagnosis [M_IQ_ = 112.23 (verbal)]	SD	Arc's SD Scale and the American Institutes for Research SD Scale	N/A	Quality of life
White et al. (37)	59	Adolescents and emerging adults (*Range*_age_ = 16–25)	Diagnosed with ASD (M_FSIQ_ = 103.36–106.39)	SD	American Institutes for Research SD Scale	The Stepped Transition in Education Program for Students with ASD	Transition readiness; Student adaptation to college
Zalewska et al. (38)	570	Young adults	Had autism as a primary disability as defined by local education agencies and based on the Individuals with Disabilities Education Act	SD	Select items from Arc's SD scale	N/A	Employment

ASD, autism spectrum disorder; SA, self-advocacy; IEP, individualized education program; SD, self-determination; FSIQ, full-scale IQ.

## 4 Results

### 4.1 SA literature

This search identified four empirical studies that represent the existing research on autistic adolescent and young adult SA related to adjustment outcomes. Various methodologies were employed to explore SA as a predictor of these outcomes, which include academic outcomes (8, 29, 32), employment outcomes (32), self-concept, friendship development (33), identity exploration, and relationship maintenance (32).

Several articles focused on the associations between SA and educational/academic outcomes, which is a crucial area of exploration for pre- and postsecondary school-aged autistic individuals. For example, Barnard-Brak and Fearon (8) used logistic regression analyses to determine that SA skills were a significant predictor of student Individualized Education Program (IEP) participation among autistic adolescents, and more so than their peers who had other disabilities. This finding is very encouraging given the positive associations between student IEP participation and academic outcomes for students with disabilities (34).

Bethune (29) tested a 7-session intervention with autistic high schoolers that taught them SA behaviors for requesting academic accommodations and conflict resolution skills. Findings demonstrated an increase in student ability to request and negotiate academic accommodations. These findings are particularly relevant for transition-aged youth, who may benefit greatly from SA skills surrounding negotiation and communication useful in future employment or educational opportunities (35).

The positive influence of SA on academic outcomes was also demonstrated by Pearlman-Avnion et al. (32) during a two-year SA preparation program designed for autistic young adults that encouraged exploration by means of experiencing different situations and opportunities. This program was highly individualized, encouraging participants to self-advocate and act autonomously in pursuit of their goals. Findings showed improvements in study skills, persistence, time management, and ultimately, postsecondary education attainment. In addition to educational benefits, the young adults in this study also showed improvements in employment attainment by the end of the first year of the SA program.

SA was also found to be positively related to several other outcomes, including self-concept, friendship and relationship development, and identity exploration. In the previously discussed study by Pearlman-Avnion et al. (32), for example, participants gained significant insight into the nature of social relationships, including friendships, relationships, and safety and sexual autonomy. In another study, Myers (33) evaluated a 6-session group SA/SD training program for autistic high school students that was found to promote positive self-concept and friendship development among a majority of the sample. Given that male self-concept and self-esteem are less consistent and show greater fluctuations than female self-concept during adolescence (36), this finding, among an entirely male sample, is encouraging. Regarding the impact of the same program's influence on friendship development, the sample reported high closeness and cohesiveness, indicating that they felt comfortable with each other and perceived the group to get along well together. On an individual level, all participants became acquaintances with two or more group members, and all relationships became closer as the study progressed. These friendship outcomes are another encouraging result from this study, as autistic individuals often experience difficulties with social interaction and in establishing friendships with their peers (3).

Taken together, the positive outcomes found across these studies have encouraging implications for the impact of SA training on educational, employment, and personal outcomes at multiple levels. Further, these studies span the adolescent, high school, and transition-aged periods, which supports prior research suggesting that that SA skills are key across multiple developmental periods (23).

### 4.2 SD literature

The search identified five studies that explore SD and adjustment outcomes among this population. These studies highlight the links between SD and several adjustment outcomes including transition readiness, college adjustment (37), employment outcomes (38), self-concept, friendship development (33), socialization (9), and quality of life (39).

Similar to the SA literature, SD was significantly associated with educational (high school and postsecondary) outcomes. The Stepped Transition in Education Program for Students with ASD (STEPS) is a 16-week program that targets SD to promote psychosocial preparedness for transition-related needs of autistic young adults, including graduation from high school and success during college education and more broadly in early adulthood (37). Results of a randomized control trial of STEPS found that high school students who completed the intervention showed significantly greater gains in transition readiness from high school that were maintained over time. Furthermore, greater SD levels at baseline were predictive of intervention gains for college adjustment. Due to its success with a population that typically does not have adequate resources to prepare them for the transition to adulthood, this intervention can be used to guide future SD intervention efforts that aim to prepare transition-aged youth for postsecondary educational opportunities. Development of SD skills are particularly important in autistic students' transition to higher education, given research showing that once enrolled in college, a significant portion of students with disabilities do not seek necessary services and supports to receive accommodations (40).

In another exploration of SD and employment outcomes in autistic young adults, Zalewska et al. (38) used the Arc's Self-Determination Scale, which is comprised of four separate subscales: personal autonomy, autonomy in career planning, self-realization, and psychological empowerment (41). Findings indicated a significant positive association only between the psychological empowerment subscale and employment outcomes, suggesting that psychological empowerment may be a particularly salient SD component for autistic young adults. The psychological empowerment subscale also corresponds to one of the SD characteristics that Wehmeyer (13) identified as important in his functional theory of SD.

SD has also been found to be associated with several intra- and interpersonal factors such as self-concept, friendship development (33), and socialization (9). As previously discussed, a combined SA/SD training curriculum for autistic high school students was found to promote positive self-concept and friendship development (33). Additionally, Lei and Russell (9) conducted qualitative interviews with autistic college students with goals of gaining insight into how these students perceived their own SD during their transition into, through, and out of college in the United Kingdom. Interviews were coded with attention to whether students described their personal experiences in a way that naturally reflected a sense of autonomy, competence and relatedness (three domains of SD according to SDT). Interview findings revealed that students purposely challenged themselves to develop socialization skills to pursue their goals of relatedness and did so by referencing their SD skills. These students also indicated that they perceived their own autonomous behaviors to result in socialization improvements over time.

White et al. (39) investigated the relationship between two complementary measures of SD, Arc's Self-Determination Scale (41) and the AIR Self-Determination Scale (42), and quality of life among autistic young adults, respectively. In this study, each measure represented different, but complementary theoretical frameworks for conceptualizing SD: the functional theory of SD (13; corresponding to the Arc SD Scale) and the self-determined learning theory (17; corresponding to the AIR SD Scale). Results indicated that both SD measures were positively associated with quality of life among autistic young adults, and those with higher SD scores reported higher life satisfaction. Taken together, these studies suggest SD-related positive outcomes in autistic samples and provide important considerations for specific SD dimensions and methods that might be utilized in future research.

## 5 Discussion

The present review attempts to elucidate the positive influence that SA and SD have on adjustment for autistic adolescents and young adults and is guided by a neurodiversity-informed lens and the notion of biosocial resilience. Based on the literature reviewed, SA and SD have the potential to positively influence several distinct domains that are particularly relevant to the adolescent and young adulthood developmental periods. These findings can be viewed in light of several limitations that inform directions for future research.

First, research on SA and SD should strive to employ more robust methodologies to supplement pilot and correlational studies that can support the ability to draw more definitive conclusions. Second, larger and more racially/ethnically diverse samples are needed to be able to generalize findings observed in studies employing small and majority White samples. Third, a systematic search strategy was not employed for this review as SA/SD research with autistic populations is still emerging and an exploratory search of the literature was deemed appropriate at this stage. Forth, future studies of SA/SD should consider strategies for recruiting and including other underrepresented autistic groups (e.g., self-identifying, women, late-diagnosed, and/or with varying communication levels). Our SAAC highlights that many of these individuals are not eligible for interventions that build SA and SD skills targeted for autistic individuals due to not having an official autism diagnosis or because interventions are not designed for the full spectrum of autism presentations. Further, cognitive and communication level were not consistently reported across studies included in this review and outdated diagnostic criteria and terminology (e.g., Asperger's syndrome, high-functioning) were often used. Future research should examine a more representative spectrum of autism that is in alignment with current diagnostic criteria (e.g., Diagnostic and Statistical Manual of Mental Disorders; DSM-5; 43). Fifth, research investigating the influence of SA and SD that incorporates a neurodiversity-affirming lens is critical, yet studies exploring these constructs with respect to autistic participants do not consistently utilize inclusive research designs (e.g., asking autistic self-advocates how the study should be designed and carried out, elevating their voices in study findings, asking for their feedback). Doing so ensures that research aligns with and is driven by values of the autistic community and shapes research initiatives in ways that are meaningful to this community, which may inherently build SA and SD as part of the research process. This review was limited in that it was not possible to obtain a complete picture of the biases that may have been present in each included study; for example, whether the interventions discussed were implemented in a neuro-affirming manner. It will be important for future studies on SA and SD to leverage participatory research frameworks where autistic self-advocates are included in the research conceptualization, design, and intervention development (44). As an example, recent research conducted by our Center and Oak Ridge Associated Universities to develop a science, technology, engineering, and mathematics (STEM) mentorship program for autistic undergraduate students utilized feedback from a steering committee comprised of autistic self-advocates that heavily influenced the direction of the study. This valuable guidance aided in the development of both the program and further research and will continue to shape this program to maximize success for transitioning these students from education to meaningful employment.

The preceding recommendations may strengthen future research on SA and SD in autism and research advocacy for autistic communities more broadly. Overall, the findings of this review present positive implications of SA and SD for the well-being of autistic adolescents and young adults, suggesting these self-processes can be fostered to build resilience by supporting autistic individuals to act with the agency and autonomy they desire.
